# Assessment of lipolysis biomarkers in adipose tissue of patients with gastrointestinal cancer

**DOI:** 10.1186/s40170-023-00329-9

**Published:** 2024-01-02

**Authors:** Federica Tambaro, Giovanni Imbimbo, Elisabetta Ferraro, Martina Andreini, Roberta Belli, Maria Ida Amabile, Cesarina Ramaccini, Giulia Lauteri, Giuseppe Nigri, Maurizio Muscaritoli, Alessio Molfino

**Affiliations:** 1 Department of Translational and Precision Medicine, Sapienza University of Rome, Rome, Italy; 2 Department of Biology, University of Pisa, Pisa, Italy; 3 Department of Surgical Sciences, Sapienza University of Rome, Rome, Italy; 4 Department of Medical-Surgical Sciences and Translational Medicine, Sapienza University of Rome, Rome, Italy

**Keywords:** Lipolysis, Cancer, Cachexia, Gene expression, Proteins

## Abstract

**Background:**

Adipose tissue metabolism may be impaired in patients with cancer. In particular, increased lipolysis was described in cancer-promoting adipose tissue atrophy.

For this reason, we assessed the expression of the lipolysis-associated genes and proteins in subcutaneous adipose tissue (SAT) of gastrointestinal (GI) cancer patients compared to controls to verify their involvement in cancer, among different types of GI cancers, and in cachexia.

**Methods:**

We considered patients with GI cancer (gastric, pancreatic, and colorectal) at their first diagnosis, with/without cachexia, and controls with benign diseases.

We collected SAT and total RNA was extracted and ATGL, HSL, PPARα, and MCP1 were analyzed by qRT-PCR. Western blot was performed to evaluate CGI-58, PLIN1 and PLIN5.

**Results:**

We found higher expression of ATGL and HSL in GI cancer patients with respect to controls (*p* ≤ 0.008) and a trend of increase for PPARα (*p* = 0.055). We found an upregulation of ATGL in GI cancer patients with cachexia (*p* = 0.033) and without cachexia (*p* = 0.017) vs controls. HSL was higher in patients with cachexia (*p* = 0.020) and without cachexia (*p* = 0.021), compared to controls. ATGL was upregulated in gastric cancer vs controls (*p* = 0.014) and higher HSL was found in gastric (*p* = 0.008) and in pancreatic cancer (*p* = 0.033) vs controls. At the protein level, we found higher CGI-58 in cancer vs controls (*p* = 0.019) and in cachectic vs controls (*p* = 0.029), as well as in gastric cancer vs controls (*p* = 0.027).

**Conclusion:**

In our cohort of GI cancer patients, we found a modulation in the expression of genes and proteins involved in lipolysis, and differences were interestingly detected according to cancer type.

**Supplementary Information:**

The online version contains supplementary material available at 10.1186/s40170-023-00329-9.

## Background

During cancer, several metabolic alterations occur and lead to peripheral tissue abnormalities, mainly represented by muscle and adipose tissue atrophy [1, 2]. Importantly, both loss of muscularity and adipose tissue have been shown to significantly impact clinical outcomes among cancer patients [3, 4].

Different metabolic alterations of the adipose tissue in cancer have been implicated in systemic alterations, including insulin resistance and inflammation [5]. Recently, adipose tissue metabolism was investigated in human cancer studies, observing increased fibrosis and inflammatory infiltration of the subcutaneous adipose tissue (SAT) [6], as well as changes in the browning process [7]. Also, enhanced lipolysis was implicated in the development of the cachexia phenotype, especially observed in experimental models [8].

Lipolysis is defined as the triacylglycerols breakdown into glycerol and free fatty acids and is promoted by adipocyte triglyceride lipase (ATGL), hormone-sensitive lipase (HSL), and monoacylglycerol lipase (MGL) [9]. This process is crucial in the maintenance of the homeostasis of lipid metabolism. Importantly, loss of adipose tissue during cancer is determined by enhanced lipolysis, altered lipogenesis, and in part by altered adipogenesis [10]. However, accumulating evidence suggests that the mechanism(s) of loss of adiposity in cancer cachexia might be mostly attributed to altered activity of the lipolytic pathways [8]. In fact, previous observations showed that reduced lipogenesis has a very limited role or no role in adipose tissue atrophy in cancer [11]. Therefore, lipolysis may play a major role in the development of involuntary body weight loss and cachexia and the modulation of lipolytic pathways may differ according to the type of cancer. In fact, data indicate different prevalence and impact of malnutrition and cachexia according to cancer site [5]. Also, there is a gap in knowledge regarding how cancer-disease-inducing cachexia may change the adipose tissue gene and protein expression. This aspect appears extremely relevant to clarify the complex pathophysiology of adipose tissue atrophy/loss in cancer.

For this reason, in our study, we aimed to investigate the changes of the main molecular markers of lipolysis in SAT obtained from patients at their first diagnosis of gastrointestinal cancers (known to be frequently associated with poor nutritional status and changes in body composition) [5, 7], as well as according to the type of gastrointestinal cancer. We then analyzed differences according to the presence of cachexia.

## Methods

### Study population

We considered a cohort of gastrointestinal cancer patients undergoing surgery for curative purposes. Inclusion criteria were a recent diagnosis of cancer (≤ 4 weeks), not receiving any anticancer therapies before surgery, age ≥ 18 years, and the ability to provide informed consent. Exclusion criteria were the presence of an ongoing acute or chronic disease negatively influencing nutritional status (e.g., chronic kidney disease, sepsis), severe cognitive impairment, or occlusion of the gastrointestinal tract. We also included in the study a control group, represented by participants undergoing abdominal elective surgery for benign conditions.

The study was conducted according to the Declaration of Helsinki and approved by the local Ethics Committee (Sapienza University, Azienda Sant’Andrea Hospital, Rome, Italy—prot. n. 167_2017). Written informed consent was obtained by all cancer patients and controls enrolled in the study.

### Clinical and nutritional variables

During the study visit, we registered among patients and controls the body weight (kg) and height (m), calculated the body mass index (BMI, kg/m^2^), usual weight, and body weight change, with a focus on non-volitive body weight loss in the previous six months. We also investigated the presence of anorexia by functional assessment of anorexia/cachexia therapy (FAACT) anorexia/cachexia subscale (A/CS) score [12]. We collected in fasting condition blood samples in EDTA tubes, and the samples were then centrifuged to further assess inflammatory and nutritional biomarkers, such as serum C-reactive protein (CRP) and albumin with standard laboratory techniques.

To determine the presence of cachexia, we referred to the diagnostic criteria of Fearon et al. [13]. In addition, during the study visit and from the patient’s clinical records, we obtained information on the comorbidities and we then collected data on the staging and histology of the gastrointestinal cancer. Also, we obtained the clinical information of the control group who underwent elective surgery.

### Adipose tissue biopsy

During the first phases of the abdominal surgery, approximately 1 cm^3^ specimen of SAT was collected. In particular, the SAT was obtained from the adipose area located in the anterior sheath of the rectus abdominis. Then, the samples collected were fresh frozen in liquid nitrogen, and stored at 80 °C.

### Adipose tissue: quantitative real-time PCR

Total RNA was extracted from subcutaneous white adipose tissue (SAT) using RNeasy Lipid Tissue Mini Kit (Qiagen, Germantown, MD, USA) according to the manufacturer’s instructions. cDNA was synthesized from 250 ng of total RNA using the High-Capacity cDNA Reverse Transcription Kits (Applied Biosystems, Thermo Fisher Scientific, Wilmington, DE, USA), according to the manufacturer’s instructions. Quantitative real-time PCR was performed with GoTaq® qPCR Master Mix (Promega, Madison, WI, USA), using Applied Biosystem 7900HT Fast. Data were normalized to β-Actin (calibrator), used as the internal control. Resulting data were analyzed using SDS2.4 Software (Applied Biosystems, Bedford, MA, USA), and fold-change was determined by using the 2−ΔΔCT, as previously described [14]. All reactions were performed in duplicate. The primers we used are shown in Supplementary Table S1.

### Adipose tissue: protein isolation and Western blotting

Total proteins were extracted from SAT by using ice-cold 2% beta-mercaptoethanol RIPA Lysis Buffer (50 mM Tris/HCl pH 8.0, 1% Triton X, 150 mM NaCl, 0.5% sodium-deoxycholate, 0.1% SDS) supplemented with Halt Protease Inhibitor (Thermo Scientific, Waltham, MA, USA) and Cocktail 2 and 3 (Sigma-Aldrich, Saint-Louis, MO, USA).

Total protein concentration was determined using Bradford protein assay (Bio-Rad, Portland, ME, USA). Proteins were fractionated by SDS-PAGE (8% and 10%) and transferred using a Trans-Blot semidry electrophoretic system (Bio-Rad, Hercules, CA, USA) to a nitrocellulose membrane according to the manufacturer’s instruction.

The membranes were incubated with primary antibodies against Comparative Gene Identification-58 (CGI-58) (1:1000) (Abcam), perilipin 1 (PLIN1) (1:500) (ProGen), perilipin 5 (PLIN5) (1:500) (ProGen), and Actin-β (1:1000) (Cell signaling) at 4 °C for 12 h. Specific antibody signals were detected by appropriate horseradish peroxidase-conjugated secondary antibodies anti-mouse (Abcam) or anti-rabbit (Abcam) or anti-guinea pig (ProGen) IgG antibody.

Immunoreactive bands were visualized by SuperSignal West Pico Plus Chemiluminescent Substrate (Thermo Scientific, Waltham, MA, USA). The relative amounts of each band were quantified by densitometric analysis using the ImageJ software. Total protein pixel intensity was normalized for those of the Actin-beta.

### Statistical analyses

Data are presented as mean ± standard deviation and median (25th and 75th percentiles), for continuous normally and non-normally distributed variables, as appropriate. Normal distribution was evaluated by the Shapiro-Wilk test. All experiments were performed at least three times. Categorical variables were shown as numbers (%). Two-tailed *t*-test or Mann–Whitney, according to normal or non-normal distribution, were performed to compare gastrointestinal cancer patients and controls, as appropriate. We evaluated differences among the type of gastrointestinal cancer (pancreatic, gastric, and colorectal) and controls, as well as among cachectic, non-cachectic, and controls by analysis of variance (ANOVA) and by the Kruskal–Wallis test, as appropriate. The chi-square test was used to verify the association(s) between categorical variables.

A *p* value < 0.05 was considered statistically significant. SPSS version 26 was used to perform statistical analyses.

## Results

### Clinical characteristics and nutritional status of the study participants

We enrolled a total of 23 patients with a new diagnosis of the following type of cancer: pancreas (*n* = 8), stomach (*n* = 7), and colorectal (*n* = 8). Cancer patients presented with a mean age of 72.1 ± 11.6 years and 12 were women (52%). The most frequent comorbidities were hypertension (61%) and diabetes (30%).

The control group had a mean age of 58.1 ± 13.9 years (*p* = 0.002), and 9 controls were women (60%) (Table 1). Cancer patients did not differ from controls in terms of BMI (kg/m^2^) (26.9 ± 3.3 vs 28.0 ± 4.3, *p* = 0.375), as well as in median C-reactive protein levels (mg/dL) (0.96 vs 0.27, *p* = 0.09).

**Table 1:** Patients’ characteristics

Parameter	Cachexia (*n* = 9)	No cachexia (*n* = 14)	Controls (*n* = 15)
Age, years	64.7 ± 13.3	76.9 ± 7.4*	58.1 ± 13.9
Males, *n* (%)	5 (56)	6 (43)	6 (40)
BMI, kg/m^2^	25.4 ± 2.9	27.9 ± 3.3	28.0 ± 4.3
Actual weight, kg	73.3 ± 13.7	78.1 ± 12.7	79.5 ± 12.2
Body weight loss in the previous 6 months, %	8.2 (6.5; 8.5)	3.4 (1.6; 4.1)^#^	0 (0; 0)
FAACT score	20.3 ± 5.9	24.8 ± 7.1	/
*Stage of the cancer disease*			
I–II	6 (67)	9 (64)	/
III–IV	3 (33)	5 (36)	/
Hemoglobin, g/dl	11.6 ± 2.5	11.1 ± 2.7	14.0 ± 2.0
Albumin, g/dl	3.3 ± 0.7	3.2 ± 0.6	3.8 ± 0.4

*Abbreviations*: *BMI* body mass index, *FAACT* Functional Assessment of Anorexia Cachexia Therapy

Variables are shown as mean ± SD and as median (inter-quartile range) for non-normally distributed values

Cachexia vs no cachexia: *p = 0.028; ^#^*p* < 0.001

Patients with cachexia were 9/23 (39%), and their median body weight loss (%) in the previous 6 months was 8.2 (6.5; 8.5). Cachexia was diagnosed in 63% of pancreatic, 29% of gastric, and 25% of colorectal cancer patients (Table 1). The control group included 15 participants undergoing surgery for non-malignant diseases, including cholecystectomy for gallstones (53%), hernia (27%), or other conditions (20%) not associated with catabolism. None of the controls referred involuntary body weight loss in the prior 6 months.

### Analysis of SAT lipolysis-associated genes and proteins of gastrointestinal cancer patients

**Fig. 1:**
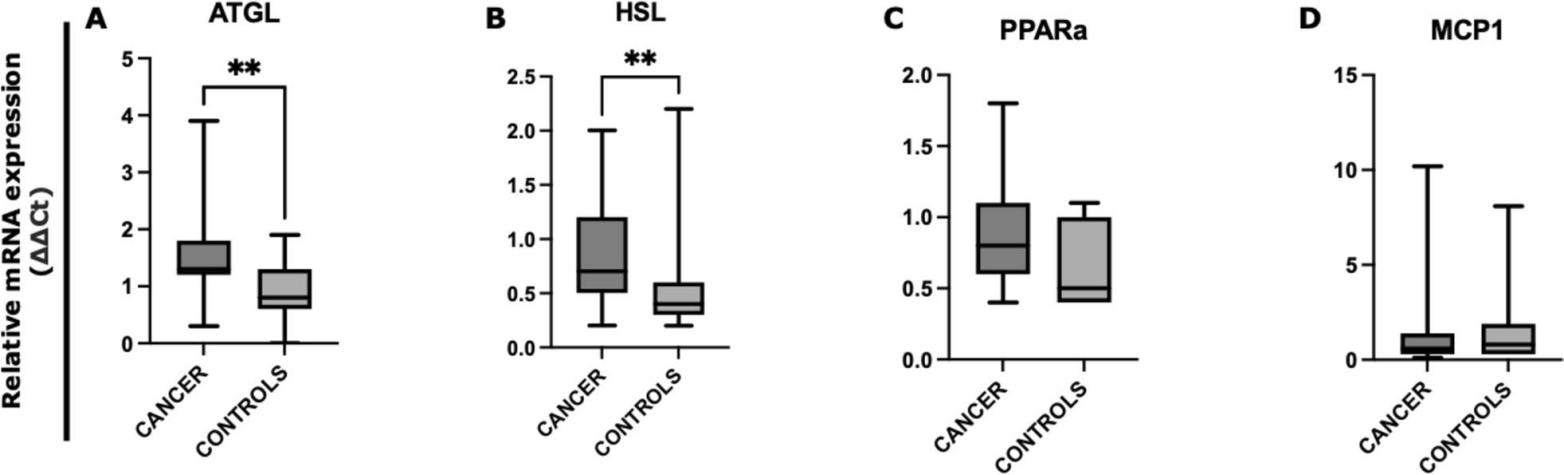
Analysis of SAT lipolysis-associated genes in gastrointestinal cancer patients and in controls. The mRNA levels of ATGL, HSL, PPARa, and MCP1 were analyzed by quantitative real-time PCR from SAT of gastrointestinal cancer patients (*N* = 23) and controls (*N* = 15). Data were normalized against the housekeeping Actin-β gene from two biological replicates and a calibrator was used as internal control. **A**, **B** Data show higher expression of ATGL (*p* = 0.008) and HSL (*p* = 0.006) in gastrointestinal cancer patients with respect to controls. **C** PPARa mRNA levels show a trend of increased expression in cancer patients compared to controls (*p* = 0.055). **D** No significant difference was observed in MCP1 mRNA levels. **p < 0.01. *Abbreviations*: adipose triglyceride lipase, ATGL; hormone-sensitive lipase, HSL; peroxisome proliferator-activated receptor alpha, PPAR-a; monocyte chemoattractant protein-1, MCP1

We examined by quantitative real-time PCR (qRT-PCR) the expression levels of SAT lipolysis-associated genes by comparing all gastrointestinal cancer patients with controls (Fig. 1). Cancer patients showed significantly higher ATGL and HSL mRNA levels vs controls (*p* = 0.008; *p* = 0.006, respectively) (Fig. 1A, B). We also observed a trend of increased mRNA levels of PPARα in cancer patients compared to controls (*p* = 0.055) (Fig. 1C), whereas no difference was found in MCP1 mRNA levels between the two groups (Fig. 1D).

**Fig. 2:**
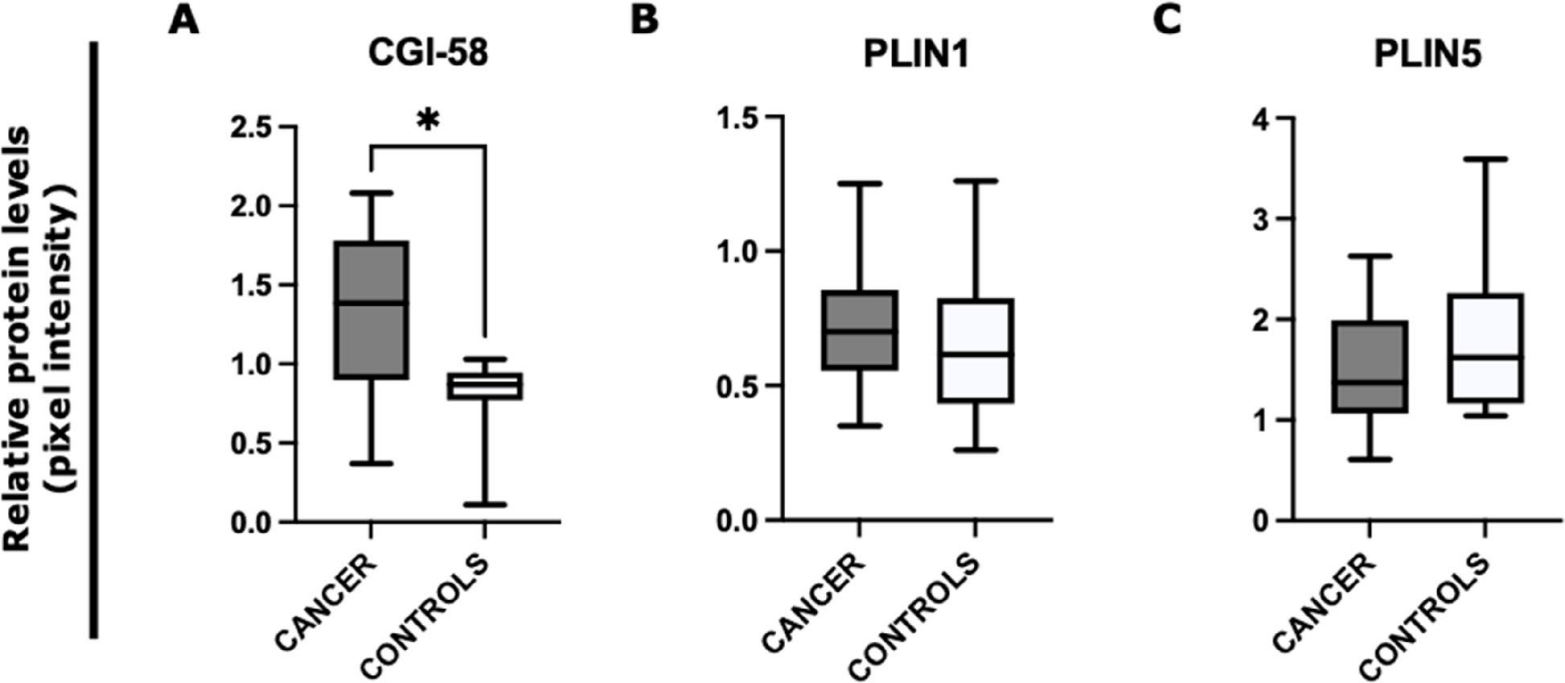
Analysis of SAT lipolysis-associated proteins by Western blot in gastrointestinal cancer patients and in controls*.* Protein densitometry quantification for CGI58, PLIN1, and PLIN5 in SAT from gastrointestinal cancer patients (*N* = 18) and control group (*N* = 8). Actin-β was used as a loading control. **A** Patients with gastrointestinal cancers showed a higher CGI58 expression level compared to controls (*p* = 0.019). **B**, **C** No significant difference was observed in PLIN1 and PLIN5 protein levels. *p < 0.05. *Abbreviations*: Comparative Gene Identification-58, CGI-58; perilipin, PLIN

In addition to gene expression, we assessed by Western blot analysis the protein levels of CGI-58, PLIN 1, and PLIN 5 between gastrointestinal cancer patients (*n* = 18) and controls (*n* = 8) (Fig. 2). We observed a significantly higher level of CGI-58 in cancer patients compared to controls (*p* = 0.019) (Fig. 2A). No significant differences were detected in PLIN1 and PLIN5 protein levels between the two groups (Fig. 2B, C).

### Analysis of SAT lipolysis-associated genes and protein levels in gastrointestinal cancer patients with and without cachexia and in controls

**Fig. 3:**
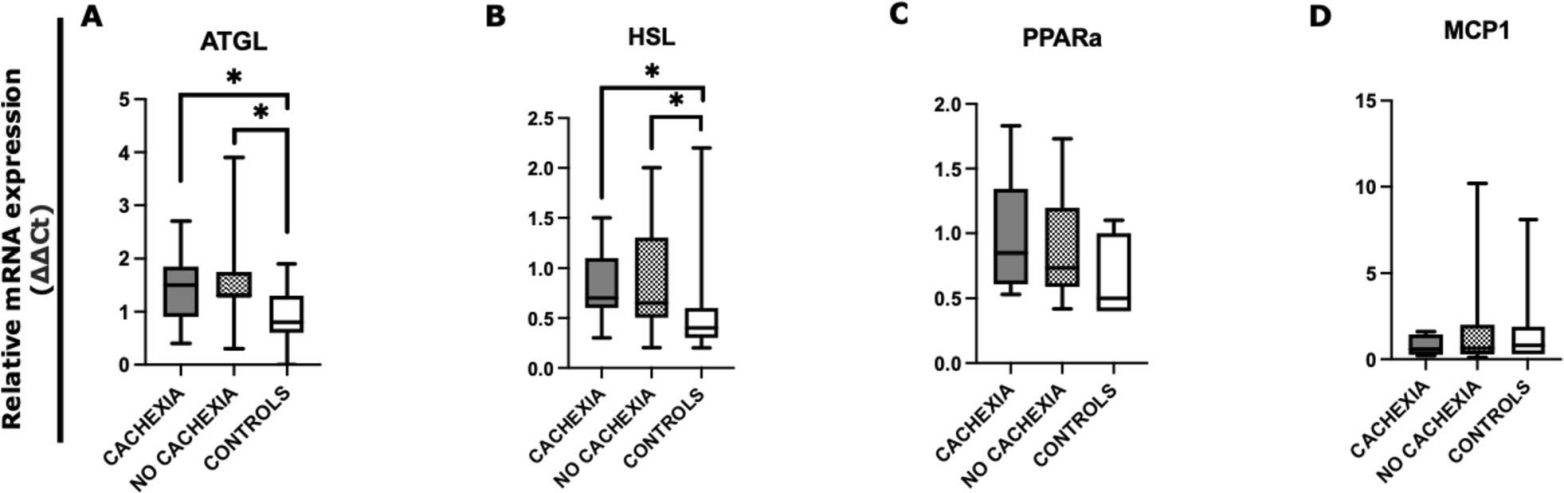
Analysis of SAT lipolysis-associated genes in gastrointestinal cancer patients with cachexia, without cachexia, and in controls*.* The mRNA levels of ATGL, HSL, PPARa, and MCP1 were analyzed by quantitative real-time PCR from gastrointestinal cancer patients with cachexia (*N* = 9), without cachexia (*N* = 14), and control group (*N* = 15). Data were normalized against the housekeeping Actin-β gene from two biological replicates and a calibrator was used as internal control. **A** Data show higher expression of ATGL in cachectic patients vs controls (*p* = 0.033) and in non-cachectic patients vs controls (*p* = 0.017). **B** HSL expression levels were higher in the cachectic group (*p* = 0.020) compared to controls and in the non-cachectic group (*p* = 0.021) with respect to controls. **C**, **D** No difference was detected in PPARa and MCP1 expression levels. *p < 0.05. *Abbreviations*: adipose triglyceride lipase, ATGL; hormone-sensitive lipase, HSL; peroxisome proliferator-activated receptor alpha, PPAR-a; monocyte chemoattractant protein-1, MCP1

We next focused on the comparison of the expression level of the same panel of SAT lipolysis-associated genes between cancer patients with cachexia (*n* = 9), without cachexia (*n* = 14), and controls (*n* = 15) (Fig. 3).

Our results showed a higher expression of ATGL mRNA levels in patients with cachexia compared to controls (*p* = 0.033) and between those without cachexia and controls (*p* = 0.017) (Fig. 3A). As shown in Fig. 3B, we observed a significant increase in HSL expression level between cachectic patients and controls (*p* = 0.020) and between non-cachectic and controls (*p* = 0.021). No significant differences were seen in mRNA levels of PPARα and MCP1 according to the presence or absence of cachexia and controls (Fig. 3C, D).

**Fig. 4:**
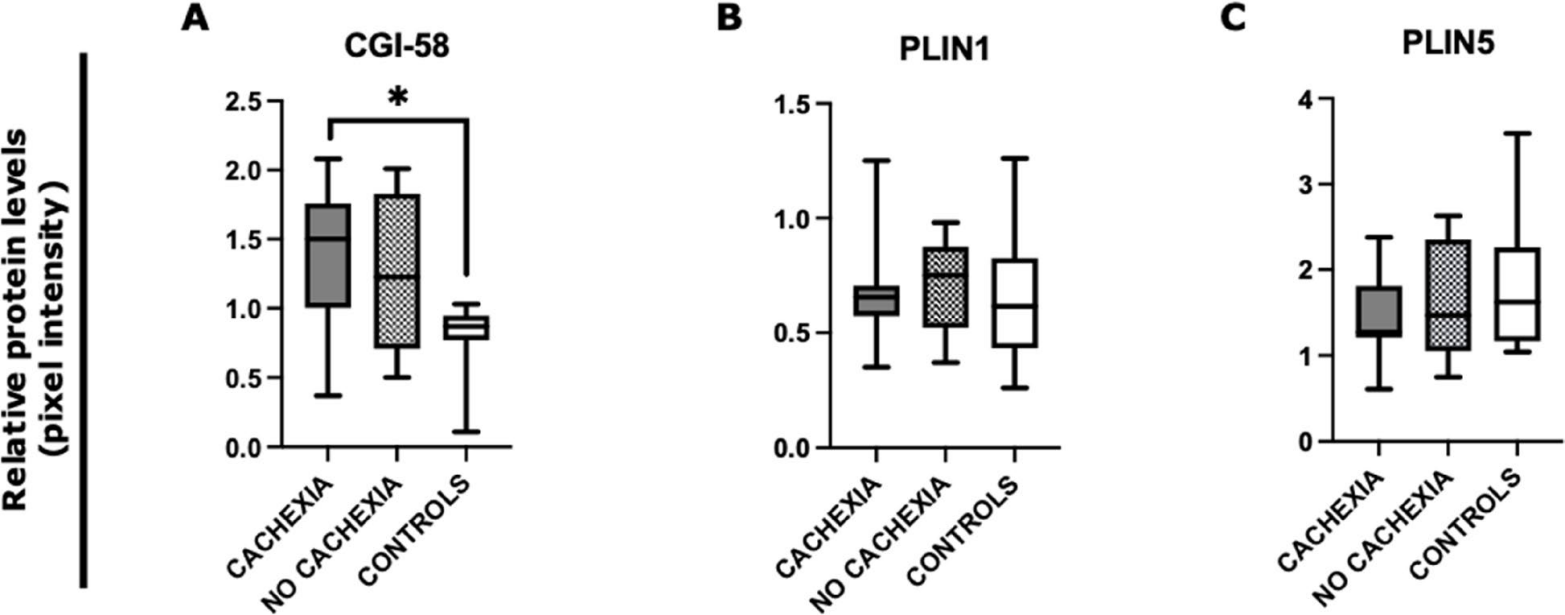
Analysis of SAT lipolysis-associated proteins by Western blot in gastrointestinal cancer patients with and without cachexia and in controls. Protein densitometry quantification for CGI58, PLIN1, and PLIN5 in SAT of cancer patients with cachexia (*N* = 7), without cachexia (*N* = 10) and controls (*N* = 8). Actin-β was used as the loading control. **A** Patients with cachexia showed higher CGI58 levels compared to controls (*p* = 0.029). **B**, **C** No significant difference was observed in PLIN1 and PLIN5 protein levels. *p < 0.05. *Abbreviations*: Comparative Gene Identification-58, CGI-58; perilipin, PLIN

By Western blot analysis, we found significantly increased levels of CGI-58 in patients with cachexia (*n* = 7) compared to controls (*n* = 8) (*p* = 0.029) (Fig. 4A). No significant difference was present in PLIN1 and PLIN 5 protein levels between cancer patients with cachexia, without cachexia (*n* = 10), and controls (Fig. 4B, C).

### Analysis of SAT lipolysis-associated genes and protein levels according to the type of gastrointestinal cancer

**Fig. 5:**
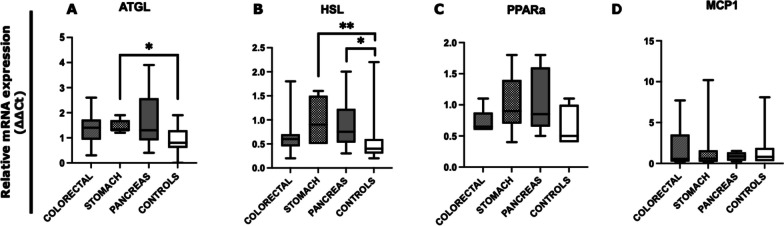
Analysis of SAT lipolysis-associated genes according to the type of gastrointestinal cancer and in controls*.* The mRNA levels of ATGL, HSL, PPARa, and MCP1 were analyzed by quantitative real-time PCR from cancer patients with colorectal (*N* = 8), gastric (*N* = 7), and pancreatic cancer (*N* = 8) and in the control group (*N* = 15). Data were normalized against the housekeeping Actin-β gene from two biological replicates and a calibrator was used as internal control. **A** Data show higher expression of ATGL in gastric cancer vs controls (*p* = 0.014). No differences were observed by comparing the other subgroups of patients. **B** HSL expression levels were higher in gastric cancer patients (*p* = 0.008) compared to controls and in pancreatic cancer patients (*p* = 0.033) with respect to controls. **C**, **D** No significant differences were observed in PPARa and MCP1 expression levels. *p < 0.05, **p < 0.01. *Abbreviations*: adipose triglyceride lipase, ATGL; hormone-sensitive lipase, HSL; peroxisome proliferator-activated receptor alpha, PPAR-a; monocyte chemoattractant protein-1, MCP1

We next analyzed data obtained by qRT-PCR according to the type of gastrointestinal cancer (Fig. 5). In particular, we found increased mRNA levels of ATGL in gastric patients compared to controls (*p* = 0.014); no significant differences were found between the other type of cancers and controls (Fig. 5A). As shown in Fig. 5B, HSL mRNA levels were significantly higher in gastric cancer patients (*p* = 0.008) and pancreatic cancer patients (*p* = 0.033) compared to controls. No differences in the other target genes of interest were found between these groups (Fig. 5C, D).

**Fig. 6:**
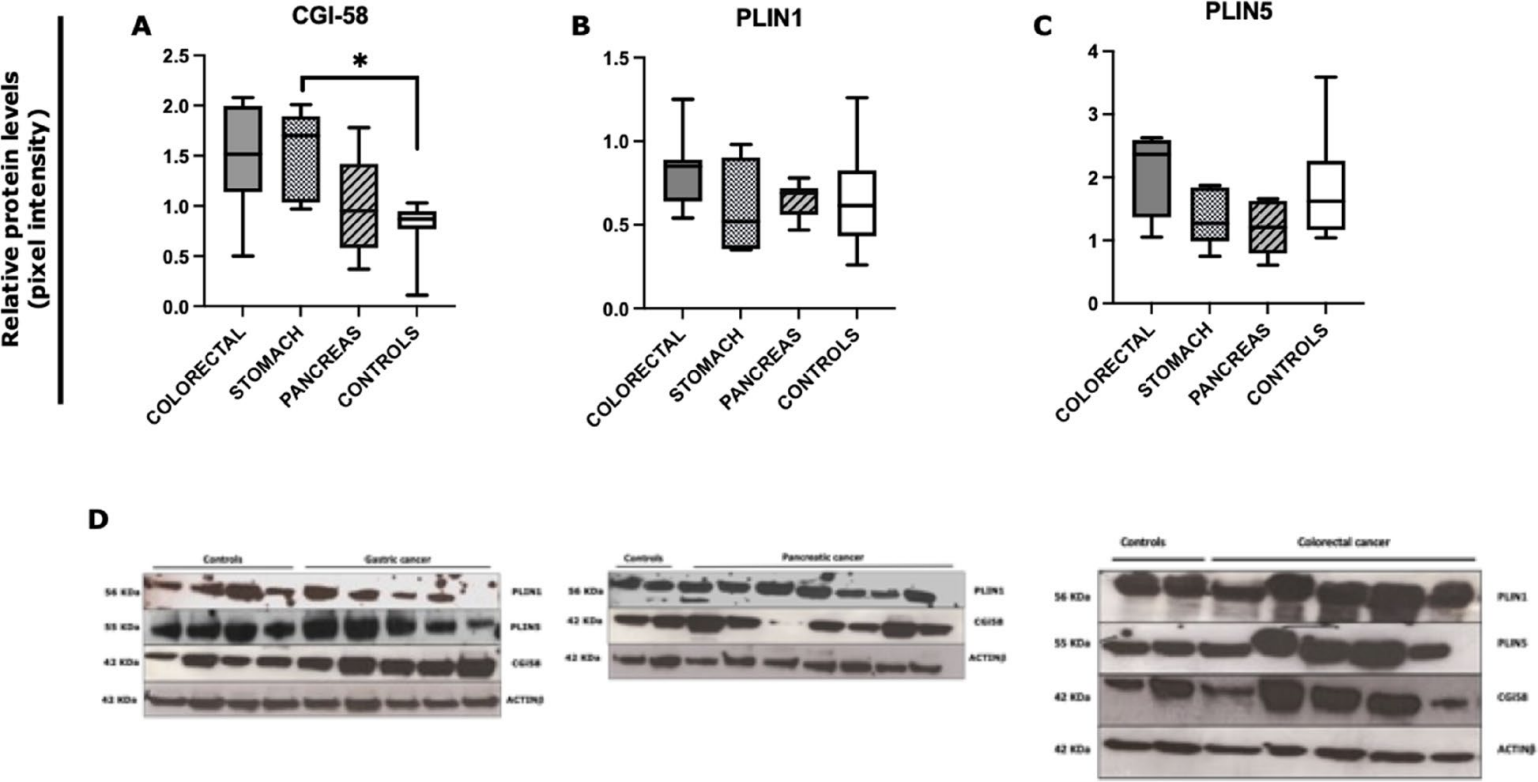
Analysis of SAT lipolysis-associated proteins by Western blot according to the type of gastrointestinal cancer and in controls*.* Protein densitometry quantification for CGI58, PLIN1, and PLIN5 with colorectal (*N* = 6), gastric (*N* = 5), pancreatic (*N* = 7) cancer, and controls (*N* = 8). Actin-β was used as loading control. **A** Patients with gastric cancer showed higher CGI58 protein levels compared to controls (*p* = 0.027). **B**, **C** No significant difference was observed in PLIN1 and PLIN5 protein levels. **D** Representative images of the SAT proteins from patients with gastric, pancreatic, colorectal cancer, and controls. *p < 0.05. *Abbreviations*: Comparative Gene Identification-58, CGI-58; perilipin, PLIN

We next analyzed the Western blot results of CGI-58, PLIN1, and PLIN5 according to cancer type (colorectal, *n* = 6; gastric, *n* = 5; pancreatic, *n* = 7) vs controls (*n* = 8). We found an increased level of CGI-58 in gastric cancer compared to controls (*p* = 0.027) (Fig. 6A). No significant differences in the other target proteins studied were found (Fig. 6B, C). Representative images of Western blotting are shown in Fig. 6D.

## Discussion

Our data indicate that in patients with gastrointestinal cancer mechanisms of lipolysis are implicated since the early phases of clinical presentation, considering that our study participants received their cancer diagnosis in the previous few weeks. In particular, we found modulation in gene expression of ATGL and HSL in SAT samples obtained from patients undergoing surgery for cancer resection. This was also documented for the expression of PPAR*α* documenting a trend of its increase in the same cohort*.* In this regard, data available in the literature indicate that adipose tissue loss is a common feature in cancer cachexia and is in part the result of deep changes in the balance of energy storage and mobilization of fat tissue [4, 15].

Mechanisms of lipolysis are tightly regulated through lipases, including ATGL and HSL [9, 16], that we tested in our cohort.

When we considered in our cancer group the presence of cachexia, we observed a greater expression of ATGL and HSL compared to controls and this was confirmed when comparing non-cachectic patients with controls, whereas no difference was present between cachectic and non-cachectic patients. In this regard, a recent study showed that adipose tissue wasting in cancer is an early phenomenon, preceding cancer clinical diagnosis by CT scan [17]. Based on this evidence, our data highlight the importance of studying and detecting molecular adipose tissue changes related to lipolysis, even before a more advanced catabolic status is clinically evident.

Our analyses showed higher expression of ATGL in gastric cancer patients versus controls, highlighting the high impact on body composition of this type of cancer, known to be frequently implicated in the consequent body weight loss and cachexia [18]. Moreover, the different type of cancer seems to affect also HSL expression, considering that patients with gastric and pancreatic cancer were those showing higher HSL mRNA levels compared to controls. Regarding PPARα, it is a well-known transcription factor belonging to the nuclear receptor superfamily and is expressed in the liver, skeletal muscle, heart, adipose tissue, kidney, and other tissues, and it plays crucial roles in fatty acid catabolism, glucose metabolism, and the regulation of energy consumption and inflammation [19]. ATGL regulates SAT lipolysis by controlling the activity of PPARα in hepatocytes [20]. Importantly, during cachexia a concomitant inhibition of adipogenesis takes place, possibly triggered by PPAR*α* [20]. In this light, our data may support this hypothesis, although we were not able to observe robust differences likely due to the small number of patients included in this analysis.

Moreover, data from the literature indicate also that cachectic, treatment-naïve patients showed increased circulating levels of MCP-1, and the authors suggested that this molecule may be useful as a biomarker of cancer-associated cachexia [21, 22]. However, in our cohort, both cancer patients and those with cachexia did not show MCP-1 modulation in SAT.

At the protein level, we also described the modulation of CGI-58 in the cancer group. The CGI-58 increase was associated with the presence of cachexia, as well as with the presence of gastric cancer. This is of particular interest, taking into account its role as a co-activator of ATGL. In fact, CGI-58 by binding ATGL promotes its activation and the consequent triglycerides hydrolysis [23].

We also studied at the protein level other key regulators of the lipolytic cascade machinery, including PLIN1 and PLIN5. In fact, the phosphorylation of PLIN1 promotes the release of CGI-58 [24] and PLIN5 has been described to modulate lipolysis and fatty acid oxidation under basal conditions [25]. In our cohorts, we did not observe significant changes in these molecules, but our results represent a novelty in the literature especially because obtained in human cancer cachexia.

We believe that the information given by our study is of interest because it integrates the understanding of the mechanisms of lipolysis associated with adipose tissue loss in cancer since the early clinical presentation of the disease in association with other clinically relevant phenomena, including adipose tissue fibrosis, inflammatory infiltration, as well as browning process [4, 6]. In fact, the molecular brakes of adiposity are complex and not yet clarified especially in humans, and in particular during the cancer journey. Moreover, a previous study showed in experimental models that enhanced lipolysis coincided with higher energy expenditure and with the browning process of white adipose tissue in an early stage of colon-26 tumor-induced cachexia [26]. In addition, Han et al. confirmed that chronic inflammation induced fat loss in cancer cachexia by promoting white adipose tissue lipolysis in the early stage of the disease in mice, as well as in human samples [27]. Similarly to our findings, other authors described that the mechanisms of increased lipolysis in cancer cachexia were mainly determined by enhanced expression and function of adipocyte HSL [28]. In this regard, adipose tissue gene expression may be modulated by both cancer and immune cells (such as macrophages and lymphocytes) through the release of cytokines or hormones, such as IL-6, TNF-α, zinc-α 2-glycoprotein, and catecholamines, which can promote lipolysis and reduce insulin sensitivity in cancer patients [29].

Moreover, our results were obtained focusing on a specific cohort of cancer patients represented by participants affected by catabolic diseases (gastrointestinal cancers) and interestingly the mechanisms of lipolysis appear modulated yet in an early phase of the disease, considering that patients were at first cancer diagnosis and eligible to surgical cancer resection. In this light, revealing pathways involved in body weight loss, even before cachexia develops, represent key factors in improving patients’ prognosis [30–32].

Our study has some limitations, including the relatively small sample size and the type of adipose tissue analyzed (SAT); more comprehensive analyses should be performed also on visceral adipose tissue, known to be implicated in several metabolic alterations [1]. However, SAT is reliable for this type of investigation in humans [33], especially for studying metabolic alterations during cancer [6, 7, 10], and is easy to obtain without major complications. The number of participants was limited especially in the subgroup of cachexia. The mechanisms of lipolysis were not integrated with the analysis of the changes of plasma fatty acids, as well as with circulating insulin levels likely implicated in the process.

We assessed the level of appetite, but we did not have information regarding energy and protein intake to be linked to lipolysis. Finally, follow-up studies should be conducted to relate the degree of body weight loss/cachexia to the degree of changes in the adipose tissue gene and protein expression.

## Conclusions

Our study revealed key molecular alterations related to adipose tissue lipolysis during an early phase of cancer disease. In particular, gene and protein expression of lipolysis of SAT were found to be modulated in a cohort of gastrointestinal cancer patients, and the upregulation of the biomarkers tested was significantly pronounced in patients with gastric and pancreatic cancer. Our data appear informative not only regarding the pathophysiology of cancer-associated nutritional and metabolic alterations but also to the implementation of anti-catabolic interventions that should be early addressed to improve the patient’s prognosis and quality of life.

### Supplementary Information

**Additional file 1:** **Supplementary Table S1.** Primers utilized for comparative real-time PCR.

## Data Availability

No datasets were generated or analysed during the current study.
