# Supplementary material for: Assessment of lipolysis biomarkers in adipose tissue of patients with gastrointestinal cancer
Source: Cancer Metab. 2024 Jan 2;12:1. doi: 10.1186/s40170-023-00329-9 (PMC10762976; doi:10.1186/s40170-023-00329-9)
Supplement: Supplementary file 1 — Additional file 1: Supplementary Table S1. Primers utilized for comparative real-time PCR. [file 40170_2023_329_MOESM1_ESM.docx]

**Supplementary Table 1.**

Primers utilized for comparative real-time PCR.

| ACTINβ | Fw 5’-CCTGGCACCCAGCACAA-3’ |
| --- | --- |
|  | Rv 5’-GGGCCGGACTCGTCATA-3’ |
| ATGL | Fw 5’-TCCTCGGCGTCTACTACGTC-3’ |
|  | Rv 5’-CTCAATGAACTTGGCACCAG-3’ |
| HSL | Fw 5’-AACTGCCAGCTGCCTTAAAA-3’ |
|  | Rv 5’-TTCCCTCACGGGAGATATTG-3’ |
| PPARα | Fw 5’-ACGATTCGACTCAAGCTGGT-3’ |
|  | Rv 5’-GTTGTGTGACATCCCGACAG-3’ |
| MCP1 | Fw 5’-AGGTGACTGGGGCATTGAT-3’ |
|  | Rv 5’- GCCTCCAGCATGAAAGTCTC -3’ |

**Abbreviations:** Adipocyte Triglyceride Lipase, ATGL; Hormone Sensitive Lipase, HSL; Peroxisome Proliferator-Activated Receptor alpha, PPARα; Monocyte Chemoattractant Protein-1, MCP1.
